# Retraction: An aberrant phosphorylation of amyloid precursor protein tyrosine regulates its trafficking and the binding to the clathrin endocytic complex in neural stem cells of Alzheimer's disease patients

**DOI:** 10.3389/fnmol.2023.1286532

**Published:** 2023-09-07

**Authors:** Frontiers Editorial Office

The journal retracts the 15 March 2017 article cited above.

Following publication, concerns were raised regarding the integrity of the images in the published figures, with areas of image duplication and manipulation in Figures 1B, 3A. Following a request for raw data, further issues were identified in Figures 5A, B, H. The authors failed to provide a satisfactory explanation during the investigation, which was conducted in accordance with Frontiers' policies. As a result, the data and conclusions of the article have been deemed unreliable and the article has been retracted.

This retraction was approved by the Chief Editors of Frontiers in Molecular Neuroscience and the Chief Executive Editor of Frontiers. The corresponding author Carmela Matrone did not agree to the retraction. Helle Rasmussen agreed to the retraction. The remaining authors did not respond to our correspondence.

